# A 20–44 GHz Wideband LNA Design Using the SiGe Technology for 5G Millimeter-Wave Applications

**DOI:** 10.3390/mi12121520

**Published:** 2021-12-07

**Authors:** Warsha Balani, Mrinal Sarvagya, Tanweer Ali, Ajit Samasgikar, Pradeep Kumar, Sameena Pathan, Manohara Pai M M

**Affiliations:** 1 School of Electronics and Communication Engineering, Reva University, Bangalore 560064, India; balani.warsha@gmail.com (W.B.); mrinalsarvagya@reva.edu.in (M.S.); 2 Department of Electronics and Communication Engineering, Manipal Institute of Technology, Manipal Academy of Higher Education, Manipal 576104, India; 3 MMRFIC Technology Pvt Ltd., Bangalore 560064, India; ajitsamasgikar@gmail.com; 4 Discipline of Electrical, Electronic and Computer Engineering, University of KwaZulu-Natal, Durban 4041, South Africa; 5 Department of Information and Communication Technology, Manipal Institute of Technology, Manipal Academy of Higher Education, Manipal 576104, India; sameena.bp@manipal.edu (S.P.); mmm.pai@manipal.edu (M.P.M.M.)

**Keywords:** mm-wave, LNA, SiGe HBT, 5G sub-system

## Abstract

This paper presents the design and implementation of a low-noise amplifier (LNA) for millimeter-wave (mm-Wave) 5G wireless applications. The LNA was based on a common-emitter configuration with cascode amplifier topology using an IHP’s 0.13 μm Silicon Germanium (SiGe) heterojunction bipolar transistor (HBT) whose f_T/f_MAX/gate-delay is 360/450 GHz/2.0 ps, utilizing transmission lines for simultaneous noise and input matching. A noise figure of 3.02–3.4 dB was obtained for the entire wide bandwidth from 20 to 44 GHz. The designed LNA exhibited a gain (S_21) greater than 20 dB across the 20–44 GHz frequency range and dissipated 9.6 mW power from a 1.2 V supply. The input reflection coefficient (S_11) and output reflection coefficient (S_22) were below −10 dB, and reverse isolation (S_12) was below −55 dB for the 20–44 GHz frequency band. The input 1 dB (P1dB) compression point of −18 dBm at 34.5 GHz was obtained. The proposed LNA occupies only a 0.715 mm^2^ area, with input and output RF (Radio Frequency) bond pads. To the authors’ knowledge, this work evidences the lowest noise figure, lowest power consumption with reasonable highest gain, and highest bandwidth attained so far at this frequency band in any silicon-based technology.

## 1. Introduction

Recent technological advancement has made 5G deployment a reality, promoting the growth of mobile data with a wide range of RF (radio frequency) connectivity solutions. The lower lag time in 5G networks compared to that of previous ones has brought in a paradigm transformation in consumer needs, thus enabling new applications. In addition, 5G systems will be tremendously fast—as much as 10 to 20 times faster than the fastest systems of today. The ever-increasing utilization of video in any setting, from social media posts and on-demand movies to video communications and security monitoring cameras, augments the need for speed in mobile systems. Furthermore, most microwave and millimeter-wave (mm-Wave) receivers have a low-noise amplifier (LNA) as the first component at the front ends. The LNA determines the performance of mm-Wave and RF receivers. Primarily, the LNA is responsible for amplifying a signal while injecting as little noise into the signal as possible. The silicon germanium (SiGe) heterojunction bipolar transistor (HBT) has the characteristics of producing very low noise and high gain over a wide bandwidth. Due to these characteristics, SiGe HBTs have seamlessly improved and presently compete with GaN and GaAs HEMTs for low-noise amplification. Hence, SiGe HBTs allow LNA integration on a single chip for mm-wave 5G sub-systems.

Earlier work focused on bandwidth extension techniques using shunt and series peaking inductors at the base. A bandwidth of 24–48 GHz and a noise figure of 3.1 dB with an overall power consumption of 17.6 mA were demonstrated [1]. In [2], an LNA was designed to operate from 26 to 42 GHz in 90 nm SOI (Silicon on Insulator) CMOS technology, providing a gain of 11.9 dB with 17 mA power consumption with a noise figure of 3.6 dB. A 20–44 GHz LNA was introduced [3] in 22 nm FDSOI (Fully Depleted Silicon on Insulator) process, which demonstrated a gain of 17 dB, the minimum noise figure of 3.1 dB, and overall power consumption of 20.5 mW. In [4], a 24–44 GHz LNA was designed in a 45 nm CMOS process, which provided a gain of 20 dB and a minimum noise figure of 4.2 dB, with a power consumption of 58 mW. A 20–44 GHz LNA was designed [5] in a 22 nm GF FDSOI process with a gain of 23 dB, a noise figure of 3.3 dB, and overall power consumption of 20.5 mW. Unfortunately, for a similar frequency band, all the above LNAs have either high power consumption or high noise figure (NF) or are still not competitive for wideband applications. 

In the presented LNA design, an approach consisting in reducing the base resistance (*R_B_*) and choosing the appropriate transistor size and current density is demonstrated to achieve low power consumption and low noise figure with high gain capabilities. The transistor size is the parameter that decides the overall power consumption as well as its noise performance. The methodology to reduce the base resistance and select the optimum transistor size is discussed in the following section. The presented LNA can be used to support applications falling under Ka-band, millimeter-wave receivers, and 5G communication systems [6,7,8,9,10]. The proposed LNA reports the lowest noise figure, lowest power consumption with reasonable highest gain, and highest bandwidth realized so far at this frequency band in any silicon-based technology. 

## 2. Technology and Transistor Sizing

**Figure 1:**
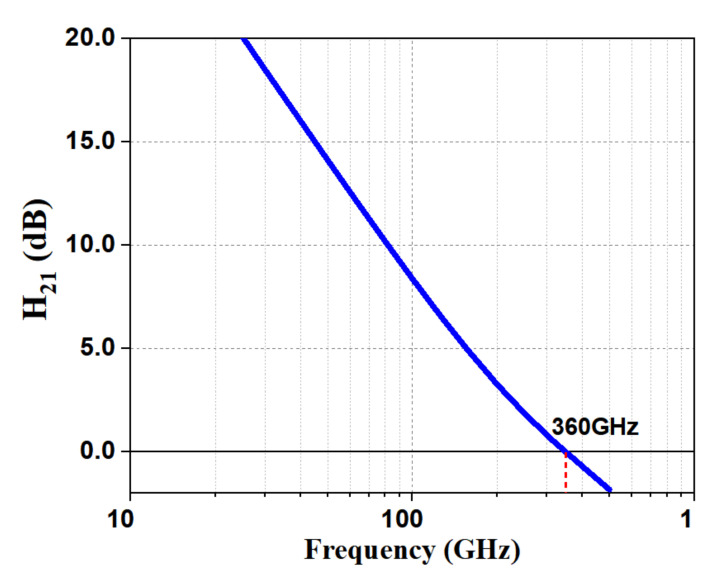
$f_{T}$ of an NPN transistor.

The presented LNA was designed in IHP’s 130 nm BiCMOS process. The technology traits high-speed HBTs with $f_{T}/f_{MAX}/gate-delay$ of 360 GHz/450 GHz/2.0 ps. The technology sets out seven aluminum metallization layers, with two thick top metal layers of 2 μm and 3 μm thickness for designing high-quality passives. As reported in Figure 1, a 1.8 μm × 0.07 μm NPN transistor achieves $f_{T}$ of 360 GHz. These features of the active device assist transistor sizing in the LNA design. 

As there is a trade-off between noise figure and gain, the appropriate transistor size needs to be selected to get the optimum values of noise figure and gain. The minimum noise figure $\left(NF_{min}\right)$ for a SiGe HBT is given by Equation (1) [11].

(1) $$NF_{min}≅1+\frac{n}{\beta }+\sqrt{\frac{2I_{C}}{V_{T}}\left(R_{B}+R_{E}\right)\left(\frac{f^{2}}{f_{T}^{2}}+\frac{1}{\beta }\right)+\frac{n^{2}}{\beta }}$$

where $V_{T}=kT/q,\;R_{B}$ is the base resistance, $R_{E}\;$ is the emitter resistance, *n* is the *I_C_* ideality factor.

For recent SiGe HBTs, Equation (1) can be simplified so that *n* is very close to 1, *ꞵ* is greater than 100, and *R_B_* is much larger than *R_E_*. Equation (1) can be rewritten as [11] (2).

(2) $$NF_{min}≅1+\sqrt{\frac{2I_{C}}{V_{T}}\left(R_{B}\right)\left(\frac{f^{2}}{f_{T}^{2}}+\frac{1}{\beta }\right)}$$

The transistor size was scaled to obtain noise matching by selecting the optimum source admittance. 

For that, initially, we determined the optimum collector current density to achieve the minimum noise figure and meet the requirements for the application in hand. From Equation (2), it can be seen that reducing *R_B_* improves the noise performance. 

For low-noise operations, the optimum source resistance is given by Equation (3) [12].

(3) $$R_{s,opt}≅\frac{f_{T}}{f}\frac{1}{\sqrt{L_{E}}}\sqrt{\frac{2R_{B}}{J_{C}W_{E}}\frac{kT}{q}}$$

From Equation (3), it is evident that *R_s,opt_*, depends on the length of the emitter (*L_E_*) and on the base resistance(*R_B_*). Most of the previously published reports focused on optimizing the current density and the length of the emitter to bring the optimum source resistance to 50 Ω, which resulted in high noise figures and high power consumption. In this paper, an effort was made to optimize the base resistance along with *I_C_* and *L_E_*. By defining additional metal layers (i.e., Metal 1 to Metal 3) on the top of the base of the transistor, we could further reduce the base resistance and hence bring the source resistance close to 50 Ω. 

**Figure 2:**
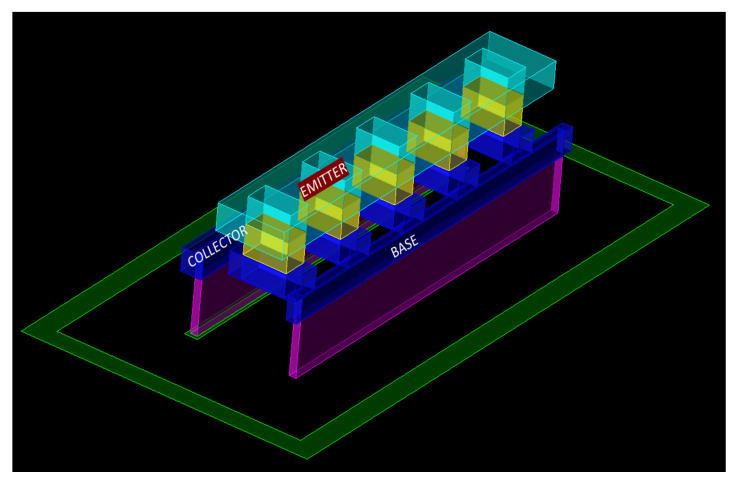
The 3D structure of the heterojunction bipolar transistor (HBT) transistor.

**Figure 3:**
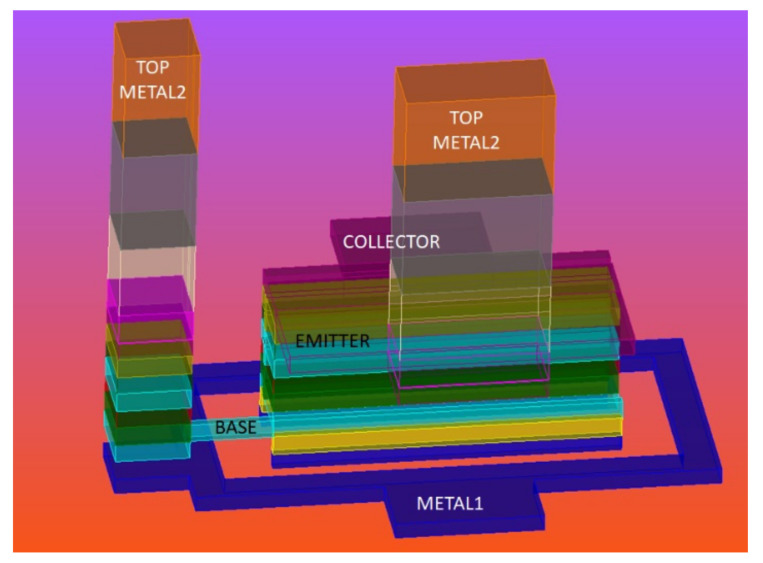
The 3D EM structure of HBT with metal stacked up on Base, Collector, and Emitter.

Figure 2 shows the 3D structure of the HBT transistor. From Figure 1, Base and Collector of the HBT were defined in Metal 1, whereas Emitter was defined in Metal 2 in the pcell structure. Each of these terminals were connected to the respective matching impedances outside the pcell. In the design, transmission lines were used for matching the impedance to 50 Ω. The transmission lines were defined in top Metal 2 layers, whereas the pcell terminals were defined in base Metal layers. Hence, it was necessary to transit from the base metal layers to the top metal layers. The transition from base metal layers to top metal layer alters the impedance matching and hence affects the overall performance of the LNA. The transition from lower metal levels to higher metal levels is unavoidable as the input RF signal always traverses from top to bottom metal levels. In order to have good impedance matching along with good performance, an effort was made to bring the real part of the input impedance close to 50 Ω before performing actual matching at the input. This was achieved by defining additional metal layers on top of the base before we physically connected the base to the transmission line. This is depicted in Figure 3. Base was defined in Metal 1, and above Metal 1, we introduced the additional Metal 2 and Metal 3 without violating any of the design rules imposed by the foundry. When these three metal layers were parallel, their effective resistance was reduced, and this in turn helped to reduce the effective base resistance. (Note: Respective via connections between the metal layers has been defined for interconnection). Now, from the HBT transistor pcell, the Base connection exited in Metal 3 instead of Metal 1. Outside the pcell, a Metal 3 to Top Metal 2 arrangement was used to connect the base to the transmission line. An EM simulation was performed in order to evaluate the effectiveness of the introduction of additional metal layers on the base. From the EM simulation, it was found that, by defining additional metal layers (i.e., Metal 2 to Metal 3) on the top of the base of the transistor, we could further reduce the base resistance and hence bring the source resistance close to 50 Ω.

**Figure 4:**
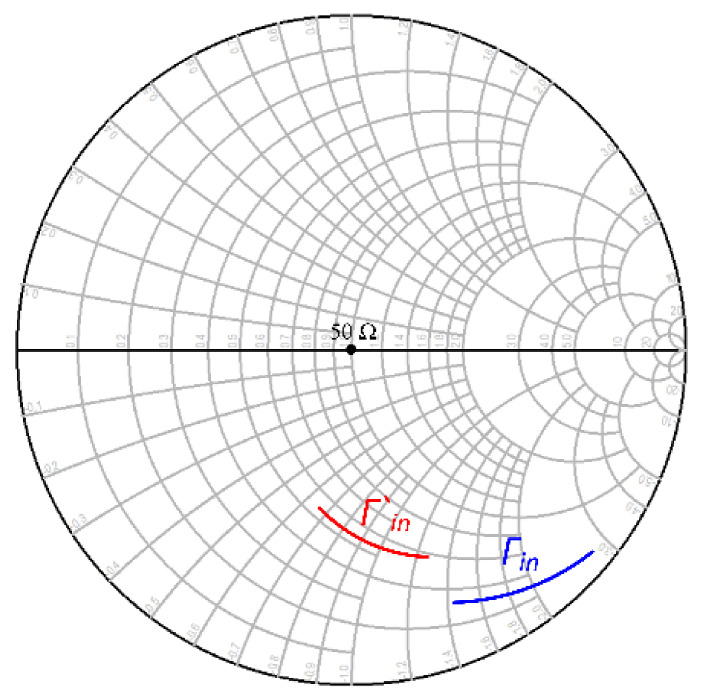
$\Gamma_{in}$ and $\Gamma {‘}_{\mathrm{in}}$ for a common-emitter (CE) common base emitter base collector (CBEBC) HBT.

An electromagnetic (EM) simulation was performed, and its performance was evaluated. Figure 4 shows the effectiveness of introducing additional metal layers on the base region in terms of reflection coefficient, which eventually resulted in bringing the source impedance to 50 Ohms. $\Gamma_{in}$ denotes the source impedance before adding any metal layers to the base region, and $\Gamma {’}_{\mathrm{in}}$ represents the source impedance after adding Metal 2 to Metal 3 layers to the base region. 

**Figure 5:**
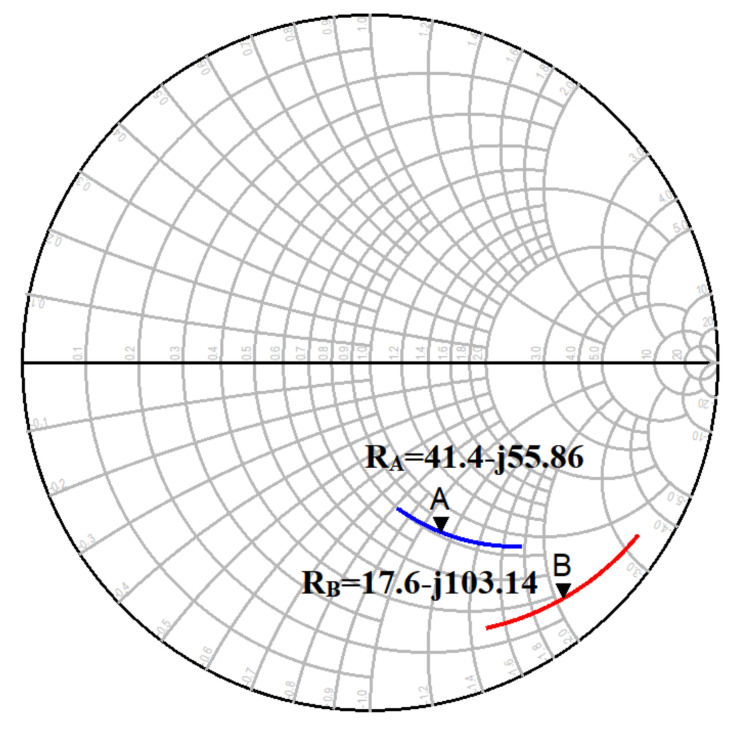
Input impedance with (A) and without (B) additional metal layers on the base region.

Figure 5 illustrates the EM simulation results. Point B (R_B_ = 17.6-j103.14 Ω @ 32 GHz) is the input impedance measured without additional metal layers on the base region, and Point A (R_A_ = 41.4-j55.86 Ω @32 GHz) is the input impedance measured with additional metal layers on the base region. From this analysis, it is very clear that the introduction of additional metal layers helped to bring the input impedance close to 50 Ω. In fact, some additional capacitance due to the introduction of metals, helped to bring the input impedance close to 50 Ω.

**Figure 6:**
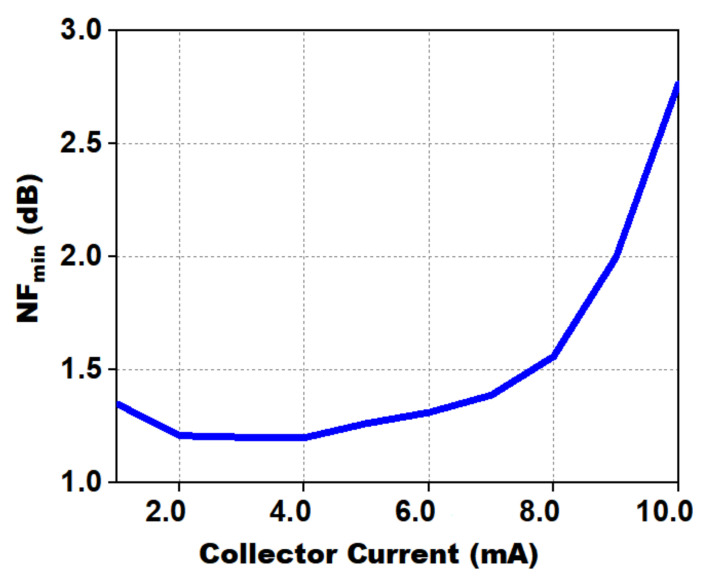
Simulated $NF_{min}$ vs. Collector Current (mA).

Figure 6 shows the primary setup used for characterization, required to ascertain the current density. NPN HBT parameterized cells (pcells) are present in IHP’s 130 nm BiCMOS SiGe technology with tunable emitter length (*L_E_*). By sweeping the collector current for a fixed emitter area, one can find the optimum current density. As shown in Figure 5, a collector current of 4 mA appeared to be an optimized value for a transistor with an emitter length (*L_E_*) of 5 μm.

The optimum transistor size and current bias were first assessed for minimum noise, maximum gain, and simplicity of matching over a wider bandwidth. In the design of the LNA, initially a parametric analysis was performed by sweeping collector current and emitter length with respect to frequency. From this analysis, we needed to find the optimum transistor size and bias current to get the minimum noise and maximum gain along, with simultaneous matching between input and output terminals.

**Figure 7:**
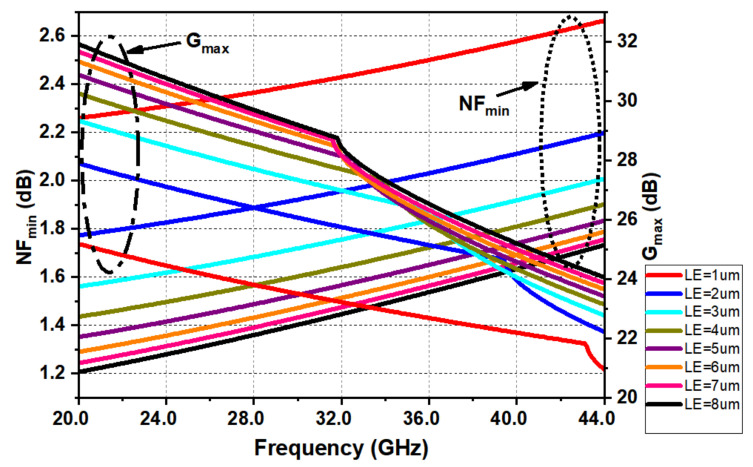
$NF_{min}$ and $G_{max}$ for a CE CBEBC HBT with emitter inductive degeneration.

A typical bias current of 0.8 mA/µm at a collector–emitter voltage of 1.2 V was initially chosen as a trade-off between maximum available gain ($G_{max}$) and minimum noise figure ($NF_{min}$). The $NF_{min}$ and $G_{max}$ for inductively emitter degenerated HBT’s over frequency versus emitter length ($L_{E}$) is shown in Figure 7.

Clearly, $NF_{min}$ was not affected as much as $G_{max}$ with respect to the device size; hence, the focus was instead placed on sizing for ease of device matching while maintaining high $G_{max}$. Once the collector current density was decided, scaling of the emitter length had to be carried out to shift the real part of the impedance to 50 Ω for noise matching. 

**Figure 8:**
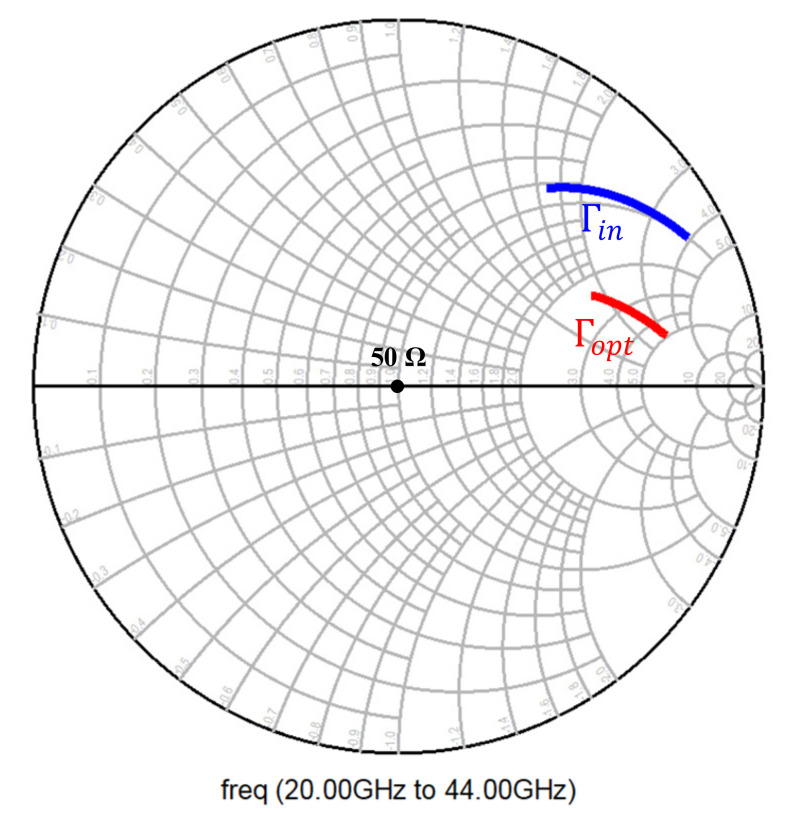
$\Gamma_{in}$ and $\Gamma_{opt}\;$for a common-emitter CBEBC HBT with inductive emitter degeneration.

To achieve this, the transistor current density was kept constant, and the emitter length (*L_E_*) of the transistor was varied to ascertain a point for which the real part of the impedance was 50 Ω for optimum noise performance. Figure 8 shows the optimal noise impedance in terms of optimal reflection coefficient for the chosen emitter length of 5 μm, which corresponded to a collector current of 4 mA.

## 3. LNA Circuit Design

**Figure 9:**
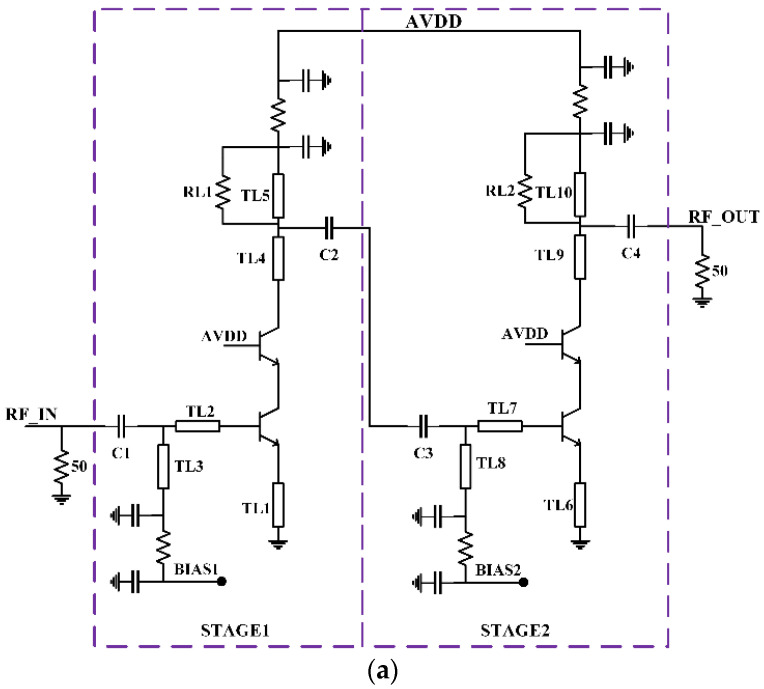

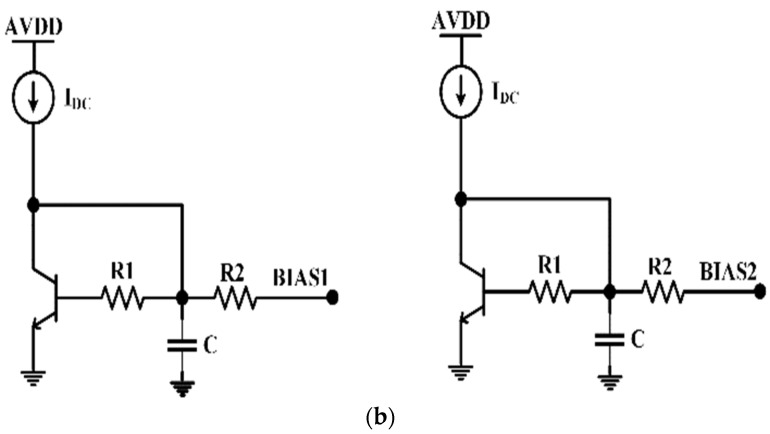
(**a**) Proposed schematic of LNA; (**b**) bias circuit.

Low power continues to be one of the primary objectives in LNA design. The ease of cascode configuration enables low-power LNAs to be employed. The presented LNA accomplishes an inductively degenerated cascode common-emitter (CE) circuit arrangement, as shown in Figure 9a. The biasing circuit used is shown in Figure 9b. Two amplification stages were incorporated in the designed LNA to realize the dynamic range with a high gain and low noise. Cascoding transistors Q1/Q2 (and Q3/Q4) were employed to impact the isolation between input and output and to minimize the effect of the parasitic base-collector capacitance of Q1/Q2 (and Q3/Q4).

The presented LNA utilizes a design procedure where the first stage is matched for minimum noise, and the second stage is matched for maximum gain. The two stages were designed such that the gain of the LNA was greater than 20 dB throughout the bandwidth of the LNA.

Input matching was primarily addressed by the series and shunt transmission lines used for biasing the base, given that the transistor size and the inductive emitter degeneration were chosen to bring $\Gamma_{in}$ and $\Gamma_{opt}\;$close to 50 Ω, as shown in Figure 8.

**Figure 10:**
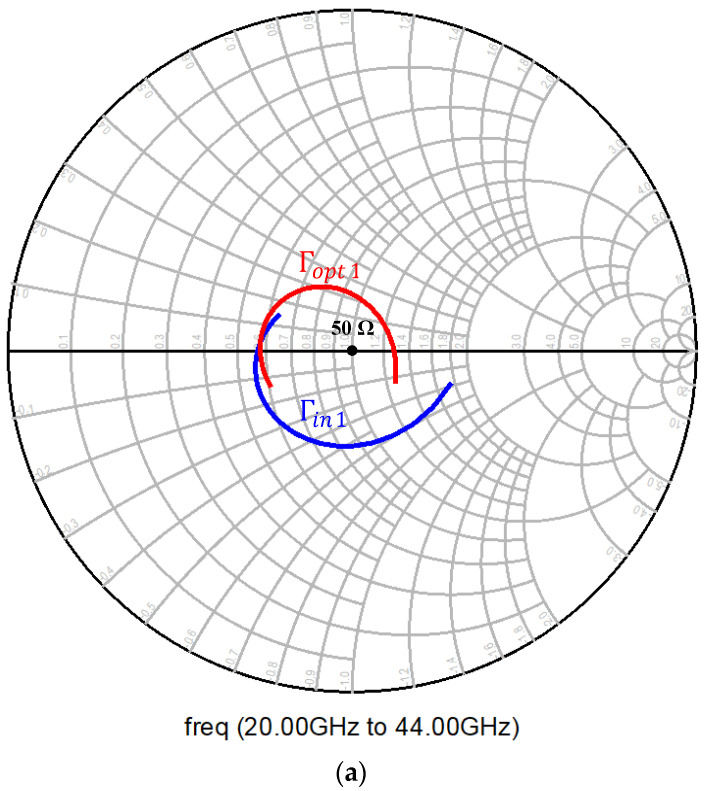

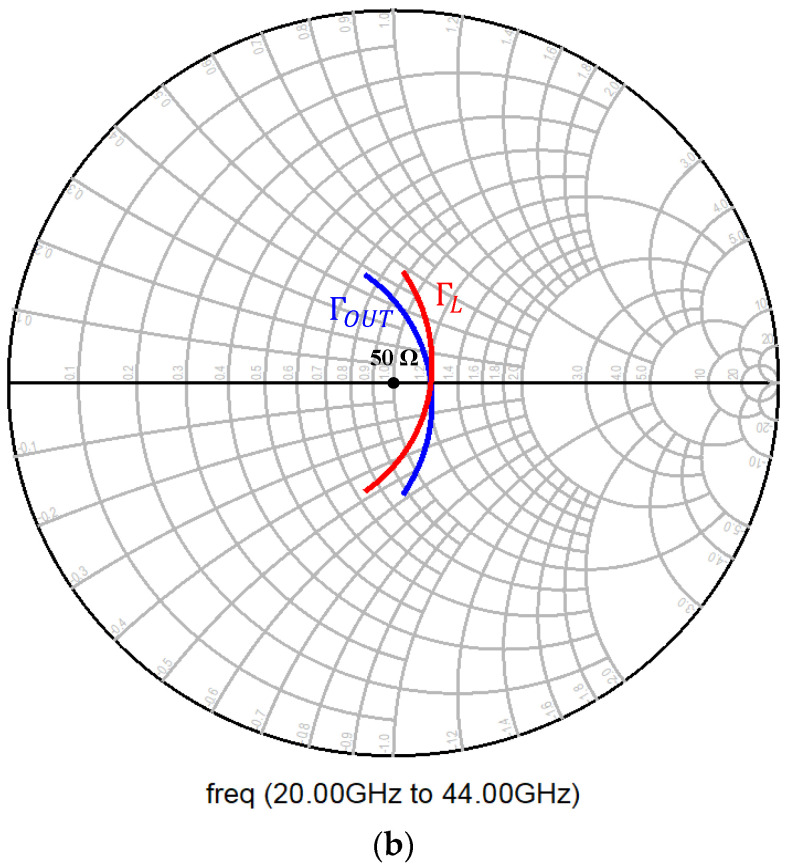
Smith chart of (**a**) input-matching network and (**b**) output-matching network over a frequency of 20–44 GHz.

As mentioned earlier, input matching was essentially addressed by the series-resonant DC block MIM capacitor, the series transmission line, and the shunt 96 Ω transmission line used for biasing the base, as the transistor sizing and inductive emitter degeneration were already selected to move the input matching close to 50 Ω. The smith chart of the input matching network is shown in Figure 10a. The input impedance of the circuit is given by Equation (4) [13,14].

(4) $$Z_{in}=-\frac{j}{\omega C_{\pi }}+j\omega L_{e}+\frac{g_{m}L_{e}}{C_{\pi }}+j\omega L_{b}$$

To match the input impedance to 50 Ω ($R_{s}$), the imaginary part Equation (4) should be equal to zero, and the real part should be 50 Ω. We can obtain the emitter and base inductor values from the Equations (5) and (6), respectively.

(5) $$L_{e}=\frac{R_{s}C_{\pi }}{g_{m}}$$

(6) $$L_{b}=\frac{1}{C_{\pi }\omega^{2}}-\frac{R_{s}C_{\pi }}{g_{m}}$$

Equations (4)–(6), provide elements to design the input-matching circuit, which is again improved by simulation of the $S_{11}$ parameter. The first stage output and second stage input were matched by incorporating interstage matching. Initially, the output impedance (Z_out1_) of the first stage and input impedance (Z_in2_) of the second stage were determined. Complex conjugate matching (Z_out1_* = Z_in2_) was incorporated between first-stage output and second-stage input [15,16]. This kind of interstage matching helped to reduce losses between the interstage and thus preserved the signal properties. 

Output matching of the stage was accomplished with a series 96 Ω transmission line and a shunt 96 Ω transmission line used for biasing the collector. The smith chart of the output-matching network is shown in Figure 10b. The biasing short stubs utilized for biasing the collector and base nodes were built by dual 2 pF shunt Metal Insulator Metal (MIM) capacitors, a 20 Ω series TaN resistor, and 500 fF of shunt MIM capacitors.

## 4. Results and Discussion

In the first stage, input-matching elements were formed by the transmission lines TL1, TL2, and TL3 and a series MIM capacitor, whereas output-matching elements were formed by the transmission line TL4 and TL5 and a series DC blocking capacitor. 

Similarly, the second-stage input-matching elements were formed by the transmission lines TL6, TL7 and TL8 and a series MIM capacitor, whereas the output-matching elements were formed by the transmission line TL9 and TL10 and a series DC blocking capacitor. All transmission lines were realized as microstrip lines, with the top metal layer (Top Metal 2) being the signal line, and the bottom metal layer (Metal 1) being the RF ground.

**Figure 11:**
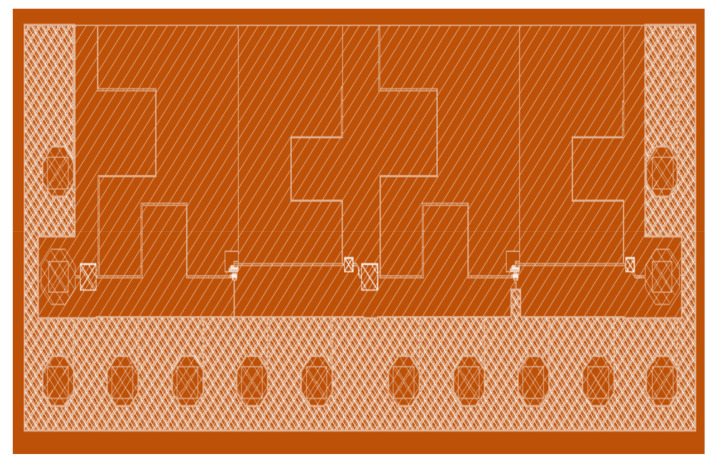
EM Layout of the two-stage LNA.

These transmission lines were synthesized and EM (electromagnetically) simulated using the Momentum tool (version 6). The EM simulation was carried out on an overall two-stage LNA layout excluding the transistors, resistors, and MIM capacitors, and an EM model was generated [7]. The EM layout model is shown in Figure 11. A test bench was created with the synthesized model of layout and plugged in with resistors, transistors, and MIM caps. The parasitic effect of RF pads was also considered while designing the respective matching sections. 

**Figure 12:**
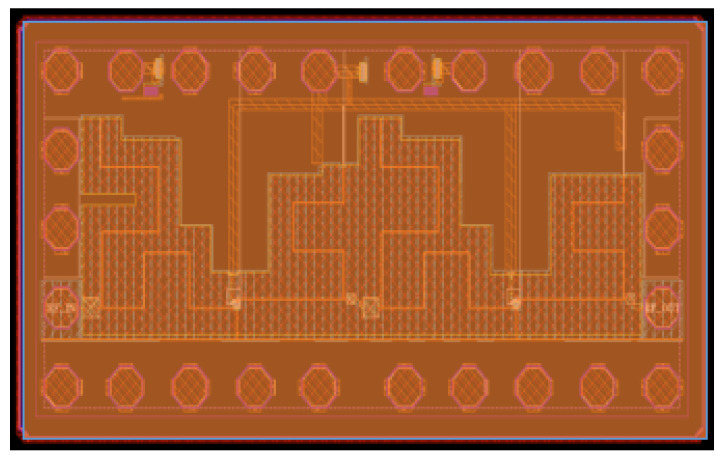
Layout of the two-stage LNA.

The complete two-stage LNA layout is depicted in Figure 12. A considerably small form factor was achieved by the use of bent transmission line structures. The presented LNA occupies a total area of 1300 μm × 550 μm with RF pads and consumes only 8 mA of total current from a 1.2 V power supply.

**Figure 13:**
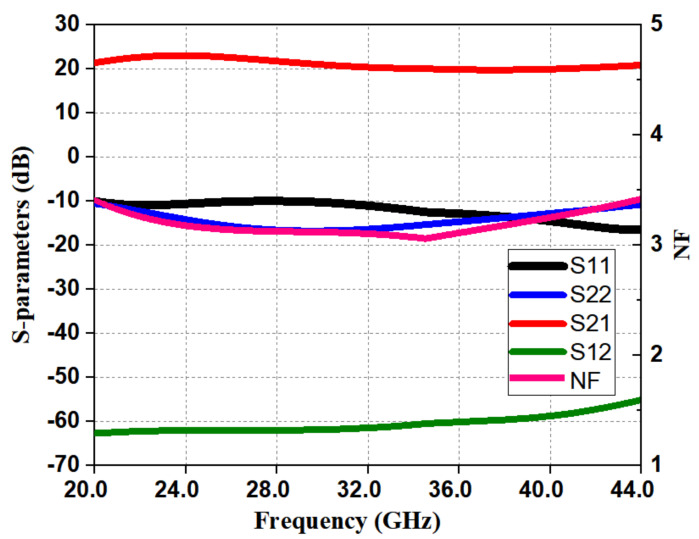
Simulated S parameters of the LNA.

**Figure 14:**
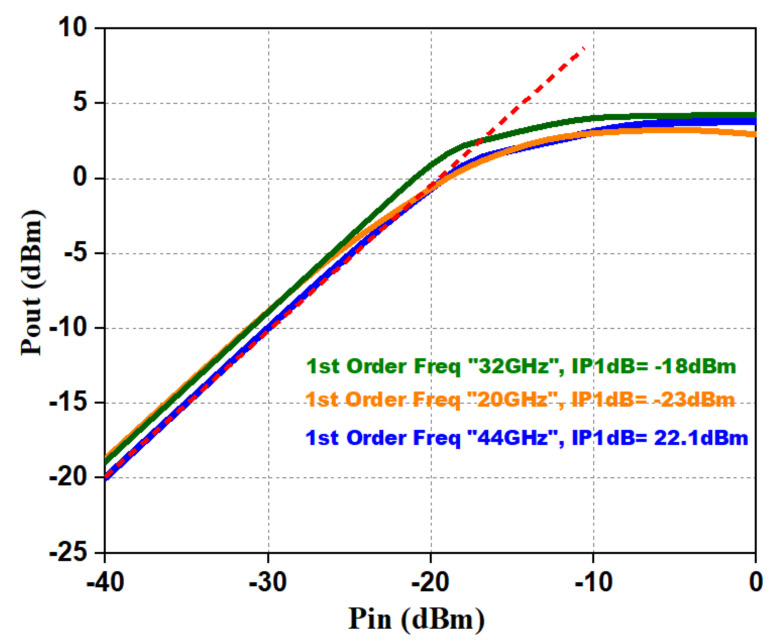
Pout vs. pin and 1 dB compression point of the LNA.

The characteristics of the LNA were validated by simulating two-port S-parameters from a frequency of 20–44 GHz. The simulated input reflection coefficient ($S_{11}$) and output reflection coefficient ($S_{22}$) are shown in Figure 13. $S_{11}$ and $S_{22}$ of <−10 dB were achieved from a frequency of 20–44 GHz with a 50 Ω source and load impedance. The simulated NF (Noise Figure) is as shown in Figure 13, and IP1dB (input 1 dB gain compression point) [17,18] is shown in Figure 14; they were 3.42 dB and −18 dBm, respectively. In Figure 13, the simulated gain appears greater than 20 dB from 20–44 GHz. The reverse isolation ($S_{12}$) of the presented LNA was < −65 dB, as shown in Figure 13.

**Figure 15:**
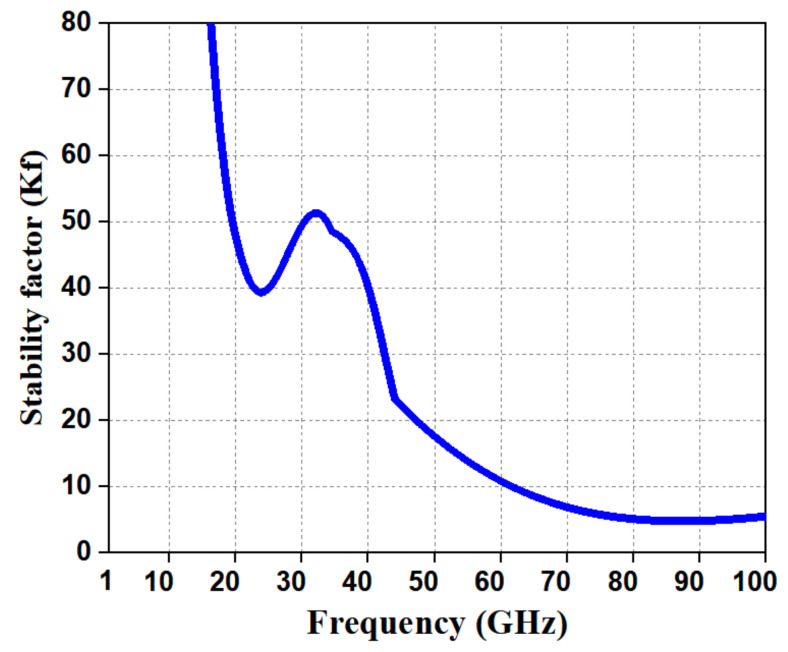
Stability factor (Kf) of the LNA.

In order to validate the stability of the LNA, a stability analysis was performed across a frequency range of 10–60 GHz. For unconditional stability, the stability factor (Kf) should be greater than 1 [19,20]. The proposed LNA was unconditionally stable in the frequency range of 10–60 GHz, which is way beyond the operating frequency range of LNAs, and its stability factor (Kf) was as shown in Figure 15.

A comparison of the LNA performance determined in this work with that from simulation tests carried out in previous publications is summarized in Table 1. The presented LNA exhibited the lowest noise figure and lowest power consumption in comparison to all previously published values. Two Figures of Merit (FoM) were defined in [6,7,8], to evaluate the performances among the wideband LNAs and are calculated using Equations (7) and (8).

(7) $$FoM_{I}=20\;log_{10}\frac{Gain\left[lin\right]\times BW\left[GHz\right]}{Power\left[mW\right]\times \left(NF\left[lin\right]-1\right)}$$

(8) $$FoM_{II}=20\;log_{10}\frac{IIP_{3}\left[mW\right]\times Gain\left[lin\right]\times BW\left[GHz\right]}{Power\left[mW\right]\times \left(NF\left[lin\right]-1\right)}$$

The input third-order intercept ($IIP_{3}$) of the presented LNA resulted to be −4.2 dBm. The $FoM_{I}$ and $FoM_{II}\;$for the proposed LNA were 26.52 and 18.11, respectively.

## 5. Conclusions

This paper details the design and simulation results of a state-of-the-art 20–44 GHz LNA circuit, implemented in a 130 nm SiGe BiCMOS technology for mm-Wave and 5G applications. A simple layout technique was adopted to minimize the base resistance of the HBT, which helped to bring the real impedance closer to 50 Ω. This design strategy was proven to be efficient to minimize the noise figure, improve the bandwidth, and lower the power consumption. The presented LNA demonstrated a gain greater than 20 dB, with a noise figure <3.4 dB in the whole 20–44 GHz bandwidth. The simulated input P1dB of −18 dBm was obtained. This work demonstrates the excellent applicability of the designed LNA for millimeter-wave applications.

## Figures and Tables

**Table 1:** Performance comparison with respect to simulated results.

Ref.	Technology	Frequency (GHz)	Gain (dB)	NF (min) (dB)	IP1 dB (dBm)
[1]	90 nm LP	24–48	Not given		---
[2]	90 nm SOI	26–42	10.5	25	---
[3]	22 nm FDSOI	24–43	19	3.5	---
[4]	45 nm CMOS SOI	24–44	18	4.2	---
[5]	22 nm FDSOI	20–44	22	4.2	−23
[21]	130 nm SiGe	55–64	24	2.5	−30
[22]	130 nm SiGe	8–12	14.5	1.4	---
[23]	130 nm SiGe	108–143	18	7.5	---
**Prop.**	**130 nm SiGe**	**20–44**	**20**	**3.02**	**−18**

## Data Availability

Not applicable.
